# A Mathematic Model That Describes Modes of MdSGHV Transmission within House Fly Populations

**DOI:** 10.3390/insects4040683

**Published:** 2013-11-20

**Authors:** Celeste R. Vallejo, Jo Ann Lee, James E. Keesling, Christopher J. Geden, Verena-Ulrike Lietze, Drion G. Boucias

**Affiliations:** 1 Department of Mathematics, University of Florida, 358 Little Hall, Gainesville, FL 32611, USA; E-Mails: cvallejo@ufl.edu (C.R.V.); joann5@ufl.edu (J.A.L.); kees@ufl.edu (J.E.K.); 2 Center for Medical, Agricultural, and Veterinary Entomology, USDA, ARS, 1600 SW 23rd Drive, Gainesville, FL 32608, USA; E-Mail: chris.geden@ars.usda.gov; 3 Entomology and Nematology Department, University of Florida, 970 Natural Area Drive, Gainesville, FL 32611, USA; E-Mail: vlietze@ufl.edu

**Keywords:** Insect virus, salivary gland hypertrophy, disease transmission, differential equations

## Abstract

In this paper it is proposed that one potential component by which the *Musca domestica* salivary gland hypertrophy virus (MdSGHV) infects individual flies is through cuticular damage. Breaks in the cuticle allow entry of the virus into the hemocoel causing the infection. Male flies typically have a higher rate of infection and a higher rate of cuticular damage than females. A model for the transmission of MdSGHV was formulated assuming several potential and recognized means of transmission. The model yields results that are in agreement with field data that measured the infection rate in house flies on dairy farms in Florida. The results from this model indicate that MdSGHV will be maintained at a stable rate within house fly populations and support the future use of MdSGHV as a birth control agent in house fly management.

## 1. Introduction 

Members within the family Hytrosaviridae are characterized as enveloped rod-shaped dsDNA viruses that infect various dipteran hosts and induce salivary gland hypertrophy (SGH) symptoms [1]. The *Musca domestica* salivary gland hypertrophy virus (MdSGHV), detected in housefly populations on a global scale, typically causes low levels of SGH [2,3,4]. Viral infection down-regulates vitellogenesis resulting in the sterilization of female flies. It is this characteristic that stimulated research addressing the potential of this virus as a birth control agent. In order to manipulate this system, mechanisms underlying virus transmission have been examined. 

Transmission of MdSGHV infection among adult house flies has been assumed to occur *per os* [5]. A typical transmission scenario would include infected flies releasing virus particles onto a food substrate that is then consumed by previously uninfected conspecifics. Upon ingestion, virus particles would bypass the midgut barriers, enter the hemocoel, and invade the target tissue, the salivary glands, for replication. Within a few days after ingestion, the newly infected flies would develop salivary gland hypertrophy (SGH) and become infectious virus donors in the population. It should be noted that once flies become infected, they remain infectious until death. Ample evidence has been provided for the presence of infectious virus particles in salivary secretions and on food substrates [2,6]. However, results from transmission experiments have raised the question whether other mechanisms that involve fly-to-fly contact are also involved in the contraction of this disease by adult flies [7]. Oral transmission experiments generally resulted in low levels of infection in the treated flies, and it was only during the initial six hours after emergence that flies were susceptible to infection [4]. Older flies, fed a single drop of virus prepared from infected salivary glands, were resistant to virus challenge. The peritrophic matrix, formed soon after emergence, likely served as a barrier to viral ingress by ingested MdSGHV (D.G.B., unpublished data). However, typically, newly emerged flies do not feed extensively during the initial six hours post emergence. Hence, we proposed that other avenues of virus transmission function in house fly populations [8]. 

Transmission by environmental contamination, for example, simulated by releasing healthy flies into cages that had previously housed infected flies, provided an additional route for MdSGHV transmission [2]. Transmission by environmental contamination is defined by contamination at gathering sites allowing for contact of deposited virus particles with the cuticle of flies. It is known that older adult flies are susceptible to MdSGHV infection by intrahemocoelic injections [5,9] and by topical applications [3]. In both cases, cuticular damage during such treatments could provide an avenue for virus particles to circumvent the peritrophic matrix barrier in the midgut and directly enter the hemocoel. 

In both field surveys and cage experiments, male flies tend to display higher levels of salivary gland hypertrophy (SGH) than females [2,7]. As suggested by Lietze *et al*. [8], the increased male susceptibility may in part be due to increased activity levels of males that potentially lead to increased cuticle wounding. It has long been noted that male flies sustain greater wing damage as they age than do female flies [10]. Female flies typically mate only once and reject subsequent male suitors who attempt copulation [11]. Courtship is initiated by males and is characterized by a sequence of physical contact that includes head lapping, head touching, and tarsal boxing [12]. The disproportionate amount of wing damage in males is due in part to mechanical injury suffered during mating attempts [13,14]. Moreover, males will also attempt to copulate with other males and are similarly injured in the process [14]. The repeated mating attempts and resulting injury to males account for most of the sex difference in longevity [15]; this is exacerbated under conditions of high fly densities or when females make up substantially less than 50% of the population [16,17]. 

Therefore, during interaction, flies, either healthy or infected, may damage the cuticle and thereby promote increased rates of MdSGHV transmission to healthy conspecifics. In this paper, we developed a mathematical model that would describe how MdSGHV may be maintained in house fly populations. Our major objective was to identify equations that would account for the differential susceptibility of males *versus* females and that would promote a stable equilibrium. The major assumption underlying this theoretical model is that the male-to-male interactions lead to increased cuticle damage that in turn result in viral ingress and viral infection. 

## 2. Materials and Methods

### 2.1. Development of the Differential Equations

**Figure 1:**
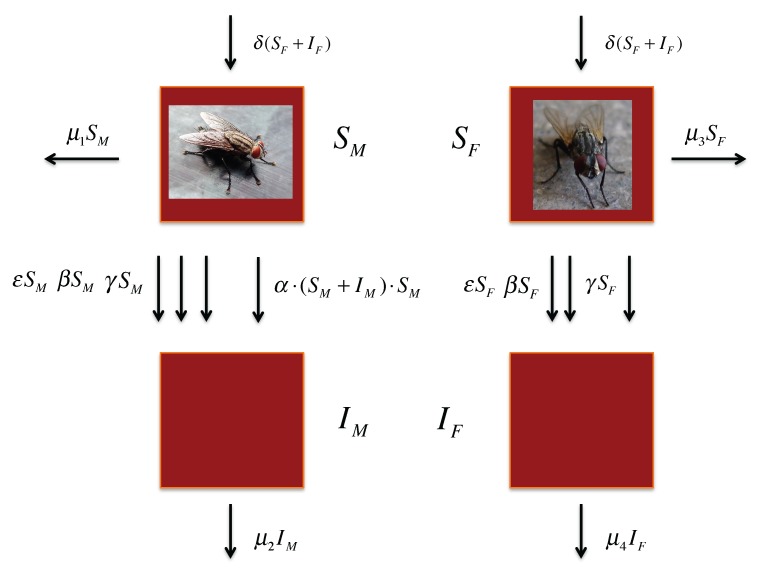
Diagram of transmission pathways of MdSGHV between infected (*I*) and susceptible (*S*) flies, where *ε* is the rate of oral infection, *α* is the rate of male interaction, *β* is the rate of transmission for feeding, *γ* is the rate of transmission for environmental contamination, and µ is the death rate for susceptible males (1), infected males (2), susceptible females (3), and infected females (4). There are no arrows from infected to susceptible because there are no instances of recovery. Images retrieved from: (http://commons.wikimedia.org/w/index.php?search=house+fly&title= Spe­cial%3ASearch).

Figure 1 gives a diagrammatic depiction of the mathematical model. The arrows between the susceptible and infected populations represent the different mechanisms for the spread of the infection that are possible in the model: infection *per os* during ingestion of food (**ε**), contamination at the feeding site followed by random cuticular damage (*β*), contamination at gathering sites followed by cuticular damage (*γ*), and interaction between males causing cuticular damage (*α*). The formulas beside the arrows indicate the rates at which infection takes place for each of these mechanisms. The top and bottom vertical arrows and the two horizontal ones show the change in the adult population due to emergence (*δ*) and death (*µ*_1_, *µ*_2_, *µ*_3_, *µ*_4_), respectively. The emergence parameter for both males and females is a function of the total number of females in the population because females give birth. Males and females are separated because male-to-male interaction introduces a quadratic term in the equations. The quadratic term reflects the greater instance of infection in males than in females. 

**Figure 2:**
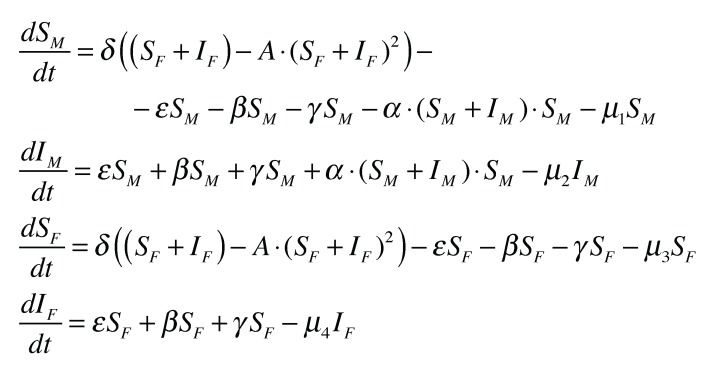
The system of differential equations derived from the diagram in Figure 1.

The diagram easily translates into a system of differential equations which are given in Figure 2 and describe the model in mathematical form. In practice, the parameters **ε**, *β*, and *γ* will vary with the level of infection by the virus in the population. However, the model assumes the sum of these to be constant, **ε** + *β* + *γ* = 0.0016. This parameter estimation was arrived at using the known values for the mortality rates and an assumed 1% infection rate. The limiting factor A reflects the carrying capacity of the population which amounts to limiting the number of susceptibles available for infection. The value for A is taken to be 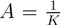, where *K* = 1,000,000. The model is expressed through four differential equations (Figure 2). 

### 2.2. Leslie Matrix for *Musca domestica* and Parameter Estimation

**Figure 3:**
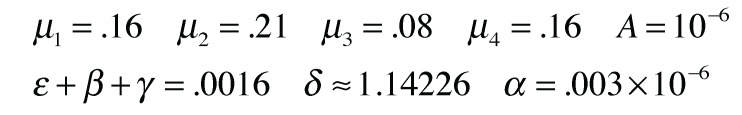
Estimated parameters of the model.

Using estimates of daily survival rates for the various stages of development in *M. domestica*, a Leslie Matrix was constructed to model the life cycle of the house fly. We assumed a 65% daily survival rate for immatures and a 91.7% daily survival rate for adults to arrive at the value for the rate of production of new adults. The model parameters were estimated based on the infection rates collected for infected flies at dairy farms in North Central Florida [2]. Estimates on the mortality rates of infected and uninfected flies were based on differences in fly longevity depending on sex and infection status, with females living longer than males and uninfected flies living longer than infected flies (C.J.G., V.-U.L., unpublished data). Estimated mortality values were:  *µ*_1_ = 0.16 (uninfected, susceptible males), *µ*_2_ = 0.21 (infected males), *µ*_3_ = 0.08 (uninfected, susceptible females), and *µ*_4_ = 0.16 (infected females). The rate of production of new adults was determined by the largest eigenvalue of the Leslie Matrix analysis using information about the mortality rates of each stage of the fly’s development. This gave *δ* = 1.14226 for the differential equations. Other rates were adjusted to give an approximate infection rate of 1%, which corresponds to the rate observed in prior field data [2]. The estimated parameters of the model are listed in Figure 3. 

## 3. Results 

### 3.1. Predictions of the Model

**Figure 4:**
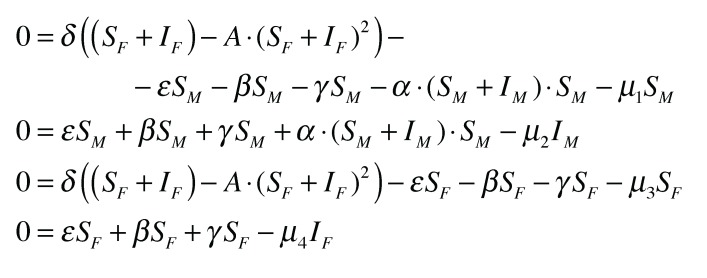
The system of differential equations at equilibrium.

**Figure 5:**
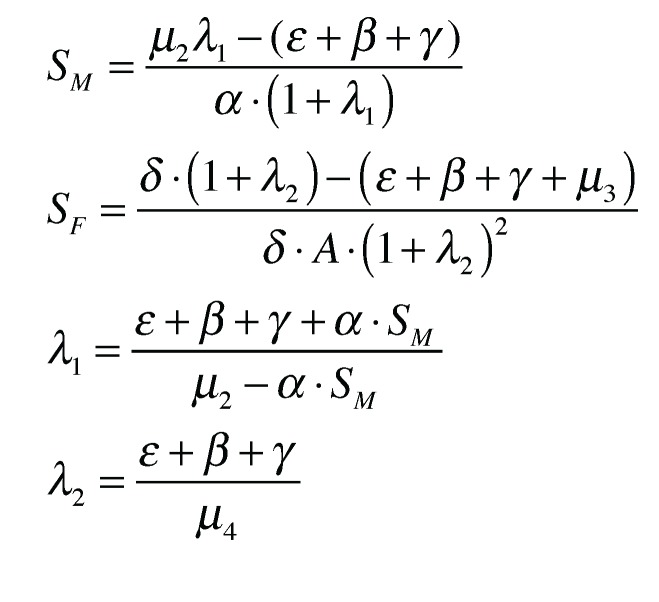
The values derived from the forced equilibrium.

The differential equations are solved numerically. We let *λ*_1_ · *S_M_* = *I_M_* and *λ*_2_ · *S_F_* = *I_F_*. The values of *λ_i_* are assumed to be approximately 1%. These are the approximate values obtained from field data [2]. The differential equations give more precise values, and the simulations converge to an equilibrium (Figure 4). Note that *λ*_2_ is constant (Figure 5). So, whatever the level of the equilibrium population, the rate of female infection will be a constant percentage of the total female population. Using the aforementioned parameters, we solve 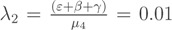. On the other hand, *λ*_1_ is not constant (Figure 5). It depends on the level of the male population, or more precisely, the density of the male population. As *S_M_* increases, so does *λ*_1_ beginning at a lowest value 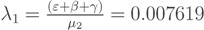.

### 3.2. The Limiting Population within the Model

**Figure 6:**
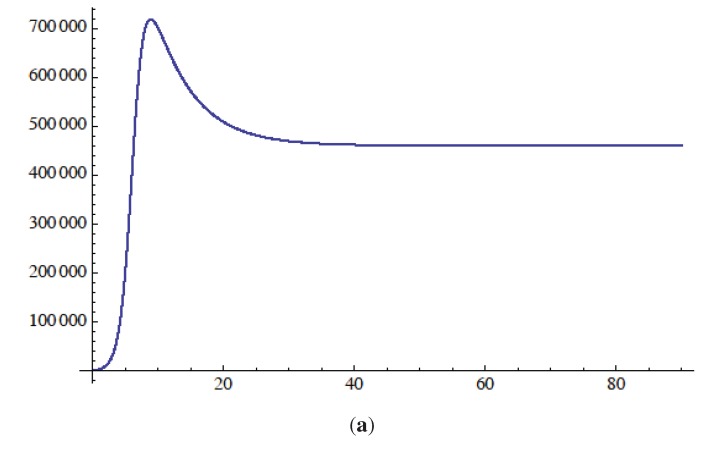

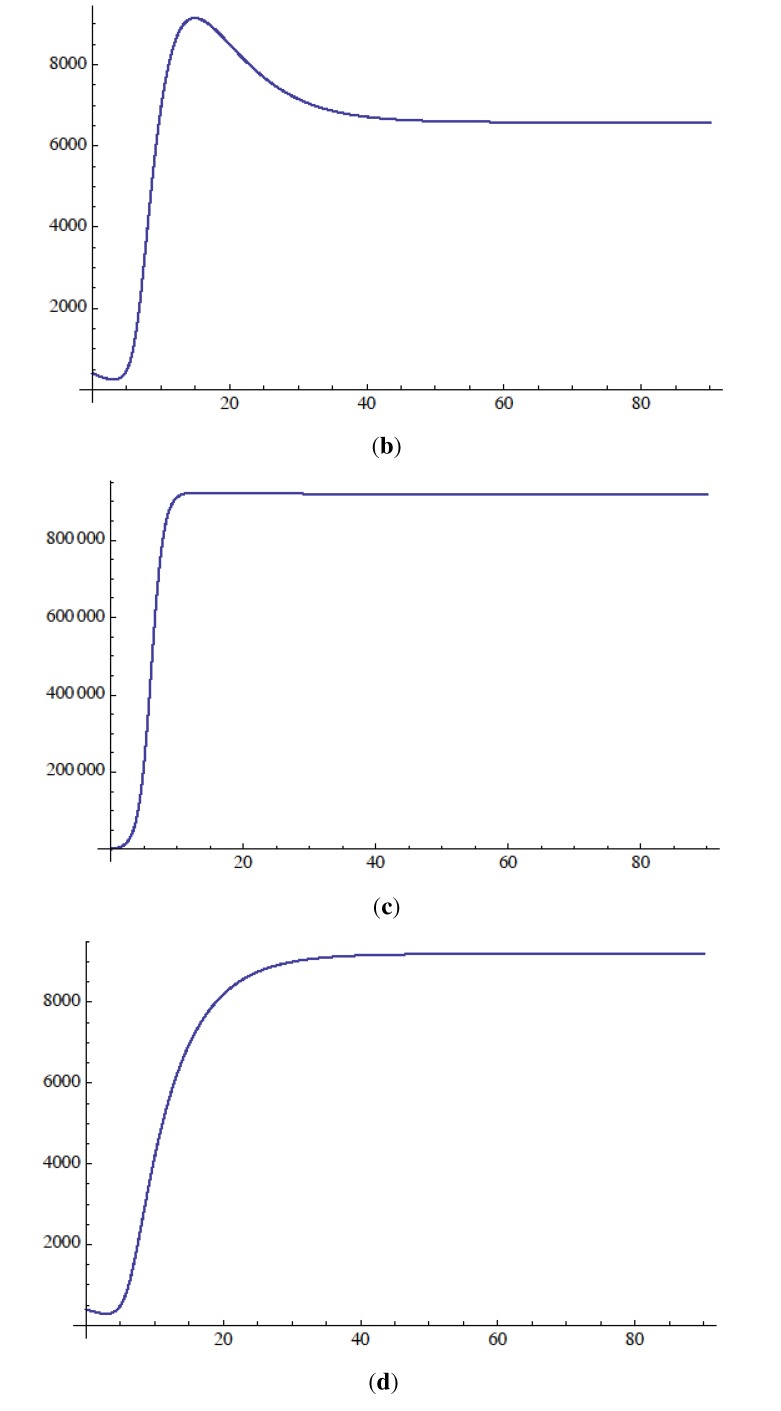
Simulations of the differential equation model. (**a**) Susceptible males; (**b**) Infected males; (**c**) Susceptible females; (**d**) Infected females. The x-axis represents time in days and the y-axis is the total population.

The model assumes a limiting population size *K*. This is expressed as a logistic equation with parameter 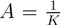. In the simulations, it is assumed that *K* = 1,000,000. This is an arbitrary parameter used in the logistic equation. In this case, it is the reciprocal of the carrying capacity for the female population. Using the previously mentioned values for parameters: *δ*, *µ*_1_, *µ*_2_, *µ*_3_, *µ*_4_, *ε*, *β*, and *γ*, and setting *α* = 0.003 · 10^−^^6^, the model was simulated for 90 days with 
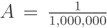
 (Figure 6). These simulations result in a 1% female infection rate and an approximate 2% male infection rate. 

## 4. Discussion 

We now describe the proposed mechanism for one component of the differential transmission of MdSGHV to male *versus* female flies in *M. domestica* adults. Healthy house flies contact the virus at sites where viremic flies have fed and rested. At the feeding sites, the enveloped virus is released through the regurgitation of crop contents onto the common feeding sites. House fly populations reach extremely high densities and display gregarious feeding behaviors. Potentially, healthy flies contaminated with viruses become infected through breaks in the cuticle allowing entry of infectious virus into the hemocoel. Prior research has demonstrated that relatively few virus particles delivered into the hemocoel can induce SGH [9]. In males, cuticular damage is assumed to occur through either male-male interactions, mating attempts, or through random cuticular injuries. Female flies, being less active (aggressive) than males, are assumed to be subjected to less cuticular damage. 

In the model, the male interaction term (*α*) is quadratic. At high population densities, this would be the principal cause of cuticular damage and would serve as a potential pathway for infection. With this assumption, the model produces higher rates of infection in males than in females. In the model, the rate of infection in females grows linearly with the size of the female population. This is approximately 1% regardless of population size. When the male population rises, the infection rate grows quadratically, is not constant, and is generally higher than the rate for females. If the model is valid, then this explains the higher rates of infection among male house flies through their behavior. Additional research is needed to confirm that the virus gains entry to the hemocoel through cuticular wounds when infected and healthy flies interact, or when two healthy flies interact with each other, since the virus may already exist on the cuticle before an interaction occurs. It seems important to determine just how cuticular damage occurs among flies, how frequently male interaction occurs, and what damage occurs during encounters. 

The model developed in this study assumes that flies enter into the population immediately in the adult stage. An improvement to the model would be to include the immature stages. This would add a lag time to the emergence of the new adults. As another correction, flies that are infected but not detected during surveys could be included in the model. Currently, the most cost-effective way to determine if a fly is infected with MdSGHV is by dissecting the fly and visually determining that the salivary glands have become enlarged. Depending on the route of infection (oral *versus* trans-cuticular) overt symptoms of infection (SGH) develop within 2 to 8 days (C.J.G., V.-U.L., D.G.B., personal observations). In programming the model, the parameter for oral infection, the parameter for feeding-site contamination, and the parameter for environmental contamination have been grouped together and assumed equal for both males and females. The result is given by *ε* + *β* + *γ* = 0.0016. Determining accurate values for these parameters and how they might vary through time would improve the model. 

In the laboratory, it is difficult to transmit the virus by force-feeding except during the first few hours after emergence [4]. However, newly emerged adults do not normally feed during this time frame. Beginning 6 hours after emergence, the peritrophic matrix enveloping the ingested material seems to be an effective barrier to infection by MdSGHV. However, in the field there may be foodstuffs that mechanically or chemically compromise the peritrophic matrix and make the oral infection pathway more likely. It should be noted that the laboratory resistance observed in older flies was recorded for adults receiving a single dose of the virus; how well this corresponds to adults continuously exposed to the various virus-contaminated food sources in nature is unknown. Making the model more accurate would involve understanding how feeding may transmit the virus under field conditions. The feeding environment is the major source of the spread of the virus in the model and represents the site where the cuticle is likely exposed to the virus. Subsequent damage to the cuticle by various occurrences allows the virus access to the hemocoel where infection takes place. 

The proposed concept of interactions amongst male house flies and their involvement in MdSGHV transmission is not unique. In various vertebrate systems, disease transmission is increased by the aggressive behaviors exhibited by males. In Norway rats, Klein *et al*. [18] found that males that were infected with the hantavirus were more likely to be aggressive. It is believed that this aggression was caused by steroid hormones (testosterone and corticosterone) that altered behaviors leading to an increased contact amongst males and resulting in higher male than female exposure to the virus. Additionally, Hamede *et al*. [19] found that aggressive behavior of male Tasmanian devils increased with an increase in population density. This agonistic behavior prompted an intensification of contact rates, thus augmenting the transmission of devil facial tumor disease in these populations. Though the above examples are vertebrate models, they serve to exemplify the way in which behavior plays a role in disease transmission. Male-male interaction behavior in house flies is one potential mechanism that results in cuticular damage that allows the virus access to the hemocoel. 

Whether or not the male-male interaction plays a role in the transmission of related hytrosaviruses is unknown. Previous reports have shown that the males of narcissus bulb flies and of various tsetse fly species display a higher incidence of SGH than females in both lab and field populations. For example, Lyon reported that field-collected males (88% SGH) had a significantly higher incidence of SGH than females (16% SGH) of the narcissus bulb fly [20]. In tsetse flies, Gouteux reported a five-fold difference in SGH expression between male and female field-collected flies [21]. It should be noted that in nature, the SGHV is vertically transmitted via milk glands or eggs in these hematophagous flies [22]. In general, these species exist at relatively low densities precluding much of the fly-to-fly interactions observed with house flies. Alternatively, it may be that the male tsetse fly is intrinsically more susceptible to virus infection than the female. This is suggested by Odindo *et al*. [23], who reported that injection of SGHV into late third instar *Glossina pallipides* led to SGH in twice as many surviving males as females. 

## 5. Conclusions 

In summary, our model suggests that transmission of MdSGHV includes *per os* acquisition, which affects female and male flies in a similar manner, and acquisition via contact with cuticle, which is more important in male infections. The developed algorithm produces simulated infection patterns that are consistent with field observations. The precise means of virus entry from contact is unknown and may include *per os* entry, entry through wounds in the cuticle, and entry through the spiracles. Further research is needed to identify whether male flies are intrinsically more susceptible to contact infection than females or whether male-specific behaviors place them at greater risk to become infected. The outcome of the model demonstrates that MdSGHV, once introduced into the population, will be maintained at a stable rate and thus may have potential as a future biocontrol agent. 
